# Mini-Review of Self-Healing Mechanism and Formulation Optimization of Polyurea Coating

**DOI:** 10.3390/polym14142808

**Published:** 2022-07-09

**Authors:** Junzhi Luo, Tao Wang, Celine Sim, Yuanzhe Li

**Affiliations:** 1 School of Materials Science & Engineering, Nanyang Technological University, Singapore 639798, Singapore; lu0003hi@e.ntu.edu.sg (J.L.); csim014@e.ntu.edu.sg (C.S.); 2 School of Telecommunications, Zhejiang College, Tongji University, Shanghai 200092, China; tjdx16060@tjzj.edu.cn

**Keywords:** polyurea coating, self-healing, self-healing mechanism, formulation optimization

## Abstract

Self-healing polymers are categorized as smart materials that are capable of surface protection and prevention of structural failure. Polyurethane/polyurea, as one of the representative coatings, has also attracted attention for industrial applications. Compared with polyurethane, polyurea coating, with a similar formation process, provides higher tensile strength and requires shorter curing time. In this paper, extrinsic and intrinsic mechanisms are reviewed to address the efficiency of the self-healing process. Moreover, formulation optimization and strategic improvement to ensure self-healing within a shorter period of time with acceptable recovery of mechanical strength are also discussed. The choice and ratio of diisocyanates, as well as the choice of chain extender, are believed to have a crucial effect on the acceleration of the self-healing process and enhance self-healing efficiency during the preparation of polyurea coatings.

## 1. Introduction

Many times, structures that require protection are in poorly accessible areas, which makes maintenance work difficult. As an industrial class of smart materials, self-healing polymers are distinguished by the fact that they can repair themselves without detection or manual intervention [1]. The concept of self-healing in polymers was introduced in the 1980s [2]. Interest in the self-healing of polymers grew when significant research progress was made by Sottos [3] in 1993 and White et al. [4] in 2001. Eventually, the European Space Agency [5] and the US Air Force [6] saw the advantages and made investments for further advances in self-healing polymers. In 2007, the first international conference on self-healing materials was held [7,8]. In recent years, studies have been conducted to equip self-healing polyurethane and polyurea with adequate mechanical properties by changing the bonds present in the polymers. The goal is to achieve an optimal balance between self-healing efficiency and mechanical properties. Lee [9] added azomethine groups to polyurethane and obtained a self-healing efficiency of 86% and a tensile strength of 50 MPa. Qian [10] introduced alkyl diselenide to polyurethane and managed to recover 100% of the polymer’s initial mechanical properties [11]. Without the need for detection or manual intervention, this can ensure that protective coatings are capable of providing continuous protection against corrosion for a prolonged time, even in the absence of short-windowed routine checks. This can also reduce the high cost of corrosion and maintenance work in poorly accessible areas. With such benefits provided by self-healing polymers, it is anticipated that the self-healing market will continue to expand significantly until at least 2025 [12].

Both polyurea and polyurethane are commonly found in coatings that protect against moisture, corrosion, abrasion, and chemicals. However, there are some differences between polyurea and polyurethane that make one a better choice over the other in different applications. Polyurea is the product of the reaction between polyamine and isocyanate (R-NCO + R’-NH_2_ → R-NH-CO–NH–R’), while polyurethane is the product of the reaction between polyol, isocyanate, and a catalyst (R-NCO + H_2_O → R-NH_2_ + CO_2_ & R-NCO + R’-NH_2_ → R-NH-CO–NH–R) [3,12]. Comparing polyurethane and polyurea, polyurea has higher tensile strength and a shorter curing time. Despite these superior properties, research in recent years has mainly focused only on the self-healing of polyurethane. Lee [9] and Hu et al. [13] used azomethine diols to achieve self-healing polyurethane elastomers with tensile strengths of at least 40 MPa. Li et al. [14] and Wang et al. [15] incorporated the Diels–Alder structure to achieve self-healing polyurethane with up to 95% healing efficiency [16]. Only limited work conducted is related to the self-healing of polyurea, and the frequency of conducting maintenance work can be greatly reduced, which makes it more capable of protecting existing structures against weathering effects [17,18] and corrosion [19]. Polyurea coatings are also known to have good corrosion resistance against seawater [20] and are capable of providing both underwater and on-land blast resistance [21] to underlying structures.

There are also varieties of formulation possibilities, incorporated with various additives, to achieve the desired performance. Much like that for polyurethane chemistry, these formulation possibilities are made possible by the selection of different types of raw materials. Thus, the selection of appropriate raw materials for the polyurea coating system can be a very complex procedure. The formulation consists of two main components (Part A: isocyanate component, reactive diluent; Part B: polyether amines, chain extenders, and additives and pigments). The most commonly used Part A components are toluene-2,4-diisocyanate (TDI) and diphenylmethane diisocyanate (MDI). TDI prepolymers with NCO content of 45 wt.% to 55 wt.% or MDI prepolymers with NCO content of 14 wt.% to 17 wt.% are preferred for standard preparations of polyurea spray coatings. In contrast, Part B, which is also named Part R, the resin blend component or polyether part, is normally a mixture of amine-terminated ethylene oxide and/or propylene oxide polyether with molecular weights varying from 200 to 5000 g/mole. Polyether amines are mainly used to increase flexibility, toughness, hydrophilicity, or hydrophobicity, and they also offer various reactivities, good thermal stabilities, colorlessness, and low viscosity.

The aim of this study is to review the self-healing mechanism and formulation optimization of polyurea coating to further accelerate the self-healing process and enhance self-healing efficiency. It aims to describe promising applications and provide practical insights into how a quickly prepared and self-healing polyurea system can further minimize downtime and maintain constant protection of substrates. The ability of polyurea to self-heal within a short period of time with the acceptable recovery of mechanical strength is important for reducing the extent of corrosion, while unwanted side reactions, such as the reduction of disulfide bonds or the saturation of hydrogen bonds, may also hinder further self-healing processes.

## 2. Self-Healing Mechanisms of Polyurea

**Figure 1:**
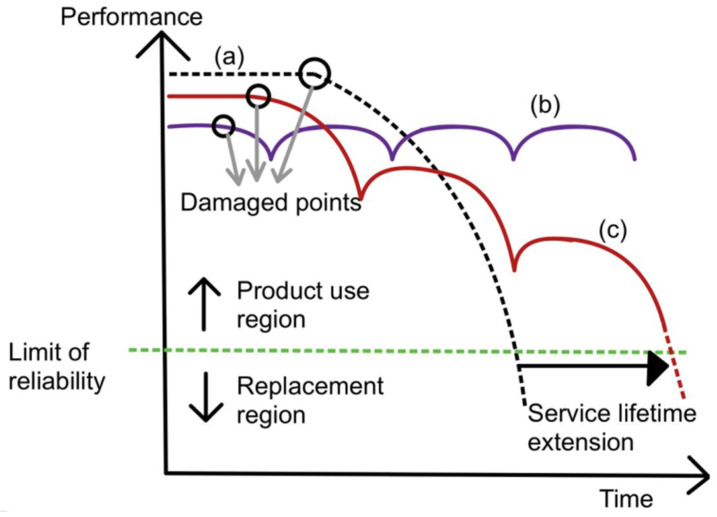
Graph of performance plotted against time for curve (**a**) (normal material), curve (**b**) (ideal self-healing material), and curve (**c**) (self-healing material) [22].

Figure 1 shows the performance of original material and self-healing material over time. Performance is defined as the original property of the material, be it tensile strength, hardness, or corrosion resistance. Service lifetime is defined as the period of time in which the material is above its limit of reliability and in working condition before it needs to be replaced. Curve (a) represents original material with a decrease in performance over time and a limited-service lifetime. Curve (b) represents traditionally improved material with a slight extension of the service lifetime. Curve (c) represents self-healing material. When there is damage inflicted on the material, its performance drops. After healing, the performance increases again and decreases due to wear over time. Once there is more damage, the cycle repeats. Due to the ability to enhance performance after healing, self-healing material is able to extend the service lifetime to a greater extent.

The working principle of polyurea self-healing mechanisms is to fill cracks by introducing more healing components, which can polymerize and seal damage in the material. Alternatively, it can also be addressed by encouraging continuous chemical reactions, which can form bonds to close gaps between the separated faces of material due to the damage.

**Figure 2:**
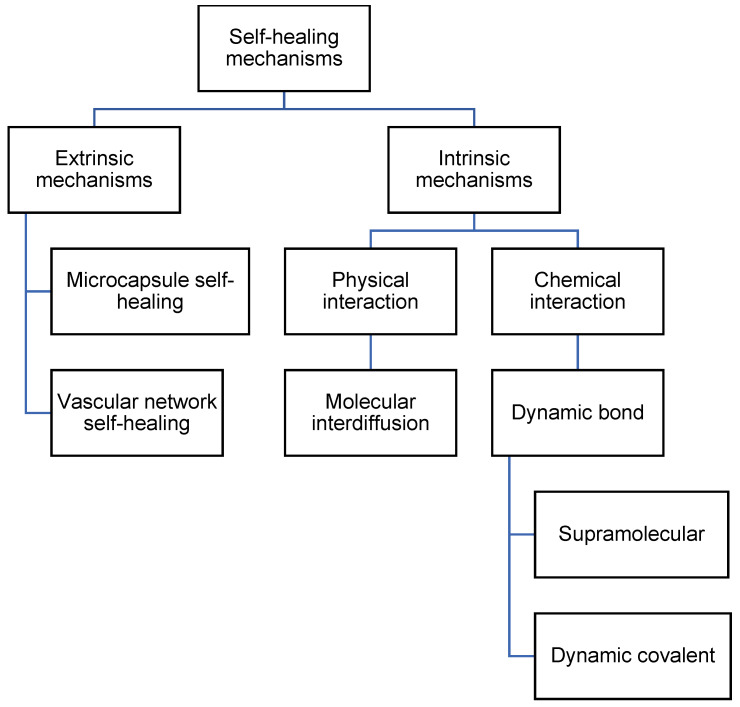
Flowchart listing the types of self-healing mechanisms in polyurea.

There are multiple mechanisms by which self-healing can occur in polyurea. Self-healing can occur by including healing agents, which are encapsulated and embedded in the polymeric matrix [4,23]. The healing agents will then be released when the encapsulation is broken. Besides that, self-healing can also occur via molecular interdiffusion [24,25], supramolecular non-covalent interaction [26], or dynamic covalent bonding [15,27]. The mechanisms of self-healing polyurea can then be differentiated into extrinsic mechanisms and intrinsic mechanisms, or autonomous mechanisms and non-autonomous mechanisms. Extrinsic and intrinsic refer to the use of additional healing agents to achieve self-healing [28]. Extrinsic mechanisms use additional healing agents, while intrinsic mechanisms do not. Autonomous and non-autonomous refer to the use of external stimuli that are not generated by damage to the material [29,30]. Autonomous mechanisms do not require the use of external stimuli for self-healing, while non-autonomous mechanisms require stimuli such as heat and light. In this review, we differentiate the mechanisms into extrinsic and intrinsic. The categorization of mechanisms is illustrated in Figure 2.

### 2.1. Extrinsic Mechanisms

Extrinsic mechanisms are defined as self-healing processes that make use of different types of containers holding prefilled healing agents that are then embedded in the matrix. Extrinsic mechanisms include microcapsule self-healing and vascular network self-healing.

#### 2.1.1. Microcapsule Self-Healing

**Figure 3:**
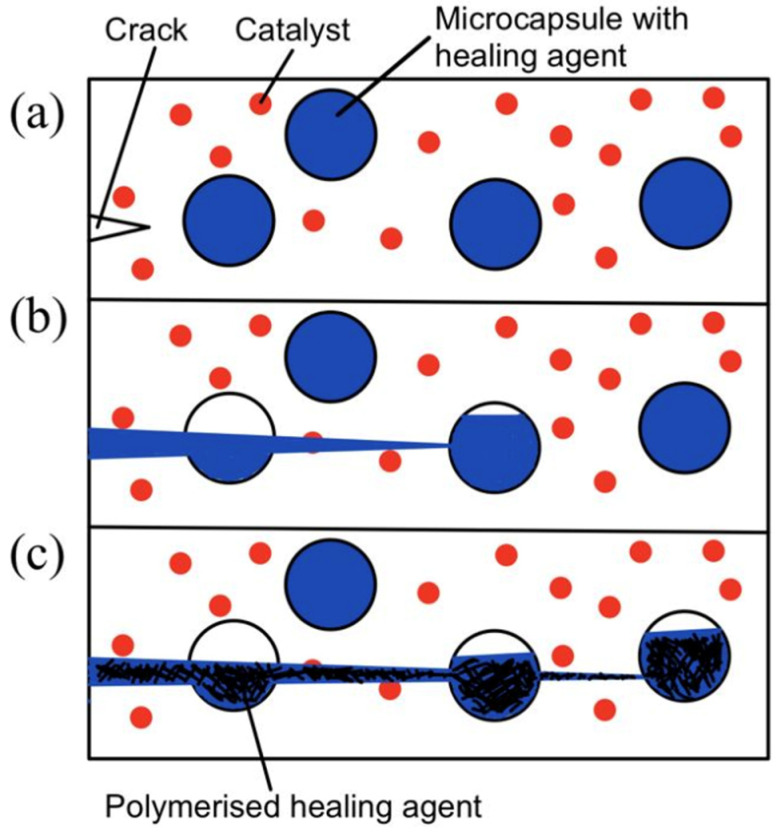
(**a**) Crack nucleation occurs in the matrix, (**b**) crack propagation takes place and ruptures microcapsules, and (**c**) microcapsules’ contents flow out and polymerize upon contact with the catalyst [4].

The microcapsule self-healing approach involves the use of healing agents that are encapsulated in capsules to heal cracks in the material. White et al. [4] prepared urea–formaldehyde microcapsules with sizes of 50–200 micrometers that are filled with dicyclopentadiene (DCPD). These DCPD-filled microcapsules are then embedded in the matrix together with Grubb’s catalyst. During service time, cracks form in the matrix. As the cracks propagate, the microcapsules rupture and release their contents. The DCPC flows out of the capsules onto the matrix. Eventually, when DCPD interacts with Grubb’s catalyst, polymerization takes place and fills the crack. Hence, self-healing occurs. This process is illustrated in Figure 3.

In recent years, multiple studies have been conducted based on this working principle [31]. However, moisture inevitably penetrates the intact coating and reacts with the healing agent. To solve the problem of the loss of healing agents due to moisture penetration, Sun et al. [32] used a double-wall microcapsule. The inner wall formed by the reaction between Tetraethylenepentamine (TEPA) and isocyanate and the outer wall formed by a physical unclonable function (PUF) constituted double-walled microcapsules. This double-wall microcapsule is proven to exhibit better water resistance and enhance long-term anticorrosion performance.

With advances in research, applications that use polyurea microcapsules have become increasingly diverse [33]. Polyurea microcapsules can be used in various areas, from self-healing anticorrosion coatings [34,35] to energy storage [36,37]. 

#### 2.1.2. Vascular Network Self-Healing

**Figure 4:**
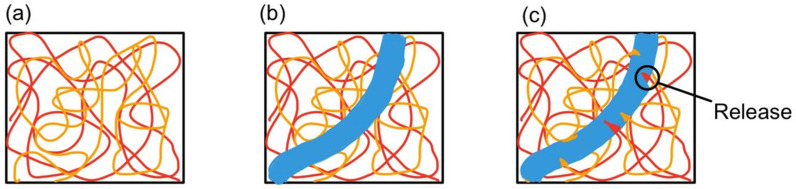
(**a**) Vascular network embedded in polymer composite matrix, (**b**) a cut is made (blue region), and (**c**) monomers from microchannel leak into the matrix [39].

Unlike the microcapsule self-healing approach, the healing agents in a vascular network self-healing system are not stored in capsules. The healing agents in a biomimetic vascular network [38] are stored in microchannels that resemble blood vessels in the human body. This vascular network self-healing approach was first demonstrated by C. Dry [23,24]. The basic working principle behind vascular network self-healing is illustrated in Figure 4.

Polymeric microchannels and a catalyst are embedded in the matrix. The monomer is then forced to infiltrate into the microchannel. Once the microchannel is damaged by crack propagation, the monomer leaks into the matrix and comes into contact with the surrounding catalyst [40]. As a result, polymerization occurs and fills the crack. Hence, self-healing takes place.

**Figure 5:**
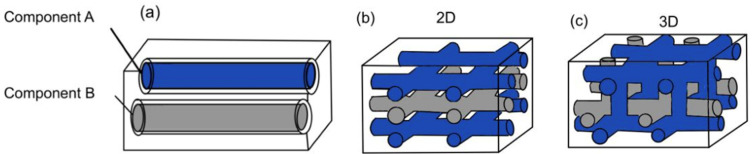
(**a**) One-dimensional vascular network system, (**b**) two-dimensional vascular network system, and (**c**) three-dimensional vascular network system [28].

Based on the way in which microchannels are embedded into the matrix, the vascular network self-healing system can be categorized into one-dimensional, two-dimensional, and three-dimensional. A one-dimensional vascular network system means that the polymer composite matrix consists of one-dimensional pipelines (Figure 5a). Both the resin and hardener are encapsulated in two different one-dimensional pipelines. Two-dimensional and three-dimensional vascular network systems have interconnected microvascular structures with both resin and hardener pipelines flowing in two dimensions (Figure 5b) and three dimensions (Figure 5c). With two- and three-dimensional microvascular structures, multiple healing cycles are made possible, as there is a constant flow of healing agents.

However, the ability of this vascular network to achieve multiple healing cycles is largely dependent on the architecture of the network [41]. As healing agents leak out from the pipeline, thrombosis of the network tends to occur. This blocks any further flow of healing agent from the pipeline to the damage site. Hence, the self-healing capability is hindered. To design a better network architecture for the enhancement of self-healing, recent studies have been tapping into additive manufacturing (AM) techniques [42,43]. AM methods such as 3D printing have been employed to produce sacrificial scaffolds to template the healing system.

### 2.2. Intrinsic Mechanisms

Intrinsic mechanisms are defined as self-healing processes that take place without the need for a healing agent or catalyst. These mechanisms are based on the inherent reversibility of the chemical bonds present in the polymeric matrix, which can be rearranged [28,39]. Intrinsic mechanisms are further divided into physical interaction and chemical interaction. With physical interaction, there is molecular interdiffusion. With chemical interaction, there is dynamic bonding.

#### 2.2.1. Physical Interaction

The phenomenon of molecular interdiffusion was first discovered when two pieces of the same polymer were brought together at temperatures above their glass transition temperatures [44]. The interfaces between the two pieces disappear, and the mechanical strength at the interface increases [44,45]. As a result, extensive research was conducted around the 1980s [25,46,47]. In particular, the Wool and O’Connor model [25] to explain the process of crack healing was more widespread. In the 1990s, research in this area slowed down [44]. The crack-healing process consists of five stages, as summarized in Table 1.

Surface rearrangement is the first step for self-healing to take place via physical interaction. Self-healing needs to take place for crack healing to occur. When the fractured surface is in contact with the healing agent, its surface topography will change with time, temperature, and pressure. This occurs due to the diffusion of chain-end distributions. The chain end can be designed accordingly to increase the efficiency of crack healing [49]. For instance, lower-surface-tension parts are applied with chain ends, and low-molecular-weight species can allow faster diffusion of chain ends from the bulk to the surface for reaction with the healing agent to heal the crack. However, oxidation and cross-linking can chemically react, disrupting diffusion kinetics. This could prevent self-healing and eventually prevent crack healing [16].

The second step is surface approach. Surface approach is the most essential step in self-healing. Surface approach must occur for self-healing [42]. The fractured surfaces have to be either brought together or surrounded by the healing agent. After the fractured surfaces approach each other, they have to wet each other to form an interface between them. The concept of wetting and the spreading of fluid was explored by Brochard [50]. As some fluids are better at wetting than others, the wettability of surfaces can determine the efficiency of self-healing [49]. The wetting stage ensures that the material has high enough chain mobility to promote diffusion in Stage 4 [42]. In a case in which the fractured surfaces have undergone chemical reactions such as oxidation, the fractured surfaces will no longer be wettable by fluid and hence will disrupt the process of self-healing.

Diffusion is the fourth stage of crack healing mechanisms. Diffusion of the chains in the polymeric matrix leads to the entanglement of polymer chains. This stage promotes the recovery of the mechanical properties of the healed material [42]. The entanglement of mobile polymer chains near the surface is also a random motion that occurs during diffusion. Hence, it is important that surface rearrangement has taken place so that chain ends are near the surface. Then, surface approach and wetting allow for diffusion across the fractured surfaces and interpenetration into the unruptured matrix material.

Randomization is the last stage of the crack healing mechanisms explained by Wool and O’Connor. This randomization stage refers to the equilibration of the non-equilibrium conformations of chains near the fractured surfaces [51]. It is also the stage where the weight distribution and orientation of chain segments near the fractured surfaces are restored.

#### 2.2.2. Chemical Interaction

Dynamic bonds refer to any type of bonds capable of undergoing repetitive breaking and reformation at an equilibrium rate [52]. Dynamic bonds can be further split into supramolecular and dynamic covalent self-healing. Supramolecular self-healing can take place at equilibrium, while dynamic covalent self-healing requires an additional intervention, such as heat or UV [28].

(a) Supramolecular

In supramolecular self-healing, non-covalent bonds and transient bonds, such as hydrogen bonds, pi–pi stacking, and metal–ligand coordination bonds, are used to generate the network. This network can then undergo repetitive breaking and reformation, allowing multiple healing events. Hydrogen bonds are capable of associating and dissociating spontaneously under ambient conditions [53]. This makes hydrogen bonds suitable for use in intrinsic self-healing materials. A hydrogen-bond-based self-healing polymer was first developed by Leibler [54], who used fatty acids and urea to design and synthesize molecules that would cross-link via hydrogen bonds. Leibler discovered that the broken samples were able to heal at a room temperature of 20 degrees Celsius until scars were invisible [55]. Upon repeated breaking and healing, the repaired sample will still break along the scar location unless a longer healing duration is permitted. As such, the sample can be broken and healed via repeated hydrogen bonding. Comparing monodentate and bidentate urea hydrogen bonds, bidentate hydrogen bonds are capable of forming stronger hydrogen linkages [56] and hence dissociate close to the polymer decomposition temperature [57].

(b) Dynamic Covalent

**Figure 6:**
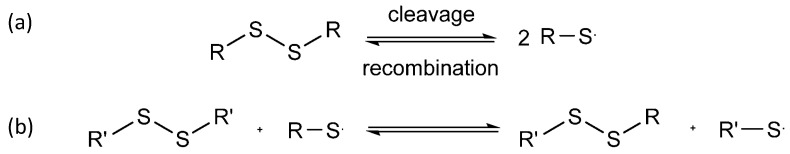
(**a**) Cleavage and recombination of disulfide bond; (**b**) 3 steps in disulfide exchange mechanism.

In dynamic covalent self-healing, covalent bonds such as disulfide bonds, Diels–Alder reactions, and imine bonds are used. Figure 6 shows the reversibility of disulfide bonds [58] and their three-step exchange mechanism [59]. Firstly, the thiol is ionized to form a thiolate anion via initialization progress. Secondly, the sulfur atom of the disulfide undergoes nucleophilic attack by the thiolate anion through propagation. This causes the cleavage of the original S-S bond and the formation of another. Thirdly, protonation may form thiol via termination. Hence, another disulfide bond is formed again.

### 2.3. Comparison between Extrinsic and Intrinsic Mechanisms

A brief summary and challenges faced when using extrinsic and intrinsic mechanisms are listed in Table 2.

Generally, microcapsules (extrinsic) face the limitation of being single-use. The vascular network (extrinsic) faces the potential blockage of core fibers due to its network, which compromises its healing properties. On the other hand, dynamic bonds (intrinsic) are capable of infinite healing cycles. This study aims to find the optimum formulation of self-healing polyurea that is capable of healing even after multiple cuts. This is to meet the practical needs of industrial uses. Hence, this study focuses on intrinsic self-healing mechanisms.

## 3. Factors Affecting Self-Healing Efficiency

Table 3 is a breakdown of the factors affecting the efficiency of both physical and chemical interactions when intrinsic self-healing mechanisms are used. In order to achieve a pre-set level of self-healing efficiency in the shortest period of time, we have to understand the factors affecting five-stage crack healing and dynamic bonds. Fast crack healing and quick reformation of dynamic bonds will lead to higher self-healing efficiency. In addition, microphase separation in the polyurea microstructure could also affect self-healing efficiency. Hence, factors that enhance self-healing efficiency include chain mobility, microphase separation, time before the surfaces are in contact, and equilibrium kinetics.

### 3.1. Chain Mobility

Chain mobility describes the ease with which polymer chains move within their matrices. According to the crack healing stages, chain mobility has to be present in Stage 1 (surface rearrangement), Stage 3 (wetting), and Stage 4 (diffusion). The greater the ease of chain mobility, the easier it is for reactive ends to react and close the crack, leading to higher self-healing efficiency. Hence, the mobility of the chain is an important factor in improving the self-healing efficiency.

Depending on the mobility of the chains, there is a maximum displacement that the broken chains can move for respective interactions to take place. The area around those chains can be differentiated into three regions [60]. The first region is where reactions between chains can take place. The second region is where reactive groups are far away, but there is a non-negligible probability of their reaction. The third region is where no reaction occurs. The number of reactive groups present in the first region will determine the self-healing efficiency.

To achieve a higher concentration of reactive groups, sufficiently high chain mobility has to be present for chains to move into the first region. Chain mobility can be enhanced by factors such as molecular weight and glass transition temperature.

#### 3.1.1. Factors Affecting Chain Mobility

(a) Molecular Weight

As the chain length increases, the molecular weight increases. The molecular weight does affect the ease of chain movement. The diffusion coefficient (1) relates chain mobility to chain length [61].

(1) $$D_{i}=\frac{k_{B}T}{n_{i}\zeta }\;$$

where $k_{B}$ is the Boltzmann constant, $n_{i}$ is the number of Kuhn segments, which relates to chain length, and ζ is the Rose friction coefficient. According to the equation, the chain mobility decreases with chain length. Since high chain mobility is favorable for self-healing efficiency, the molecular weight of the polymer chain should be reduced.

However, the mechanical properties of polymers are a function of molecular weight [62]. Mechanical properties improve with increasing molecular weight. There has to be a minimum required molecular weight to ensure the strength of polymers.

Although the self-healing efficiency of self-healing polyurea should be placed as the priority, polyurea with weak mechanical properties is of little value to industrial needs. There must be a balance between restoring cracks via self-healing and maintaining the mechanical properties of polyurea for the material to serve its purpose.

(b) Presence of Pendant Group

Given the same molecular weight, the presence of pendant groups in the components involved will affect chain mobility. There are two different types of pendant groups: bulky and flexible [63]. In the case of components with bulky pendants, such as a benzene group, the bulky pendant will hinder rotational freedom. As a result, chain mobility is restricted. On the other hand, components with flexible pendant groups, such as aliphatic chains, will limit the packing of chains. Hence, rotational motion is allowed, and chain mobility increases.

(c) Glass Transition Temperature

The glass transition temperature (T_g_) is defined as the temperature required for 30–50 carbon chains to start moving. At the glass transition temperature, amorphous regions experience a transition from a rigid solid state to a more flexible rubbery state. As a result, the free volume increases by 2.5 times [64]. With the increase in free volume, Fickian diffusion is enhanced. This diffusion is driven by reptation [1], which is the thermal motion of entangled macromolecules. Hence, Fickian diffusion helps with chain mobility and favors the self-healing process. Thus, lowering T_g_ for the material to enter the flexible rubbery state will improve chain mobility.

#### 3.1.2. Influence of Choices on Chain Mobility

The choice of diisocyanate affects the chain mobility, as the molecular weight and the presence of the pendant group vary. Thus, the glass transition temperature will also be affected, which in turn affects the chain mobility at the specific temperature.

Comparing the diisocyanates in Table 4, molecular weight increases as follows: BDI < TDI < MDI. MDI and TDI contain bulky pendant groups, while BDI does not. The rigid aromatic diisocyanates (MDI and TDI) will enhance rigidity and reduce chain mobility as compared to their flexible aliphatic counterpart (BDI). Hence, the choice of diisocyanate can affect chain mobility, which is a significant factor that determines the self-healing efficiency of polyurea.

### 3.2. Microphase Separation

Self-healing efficiency is also dependent on microphase separation. Polyurea consists of a segmented structure comprising hard and soft segments. The hard segment is made up of isocyanate and a chain extender. The soft segment is made of long-chain diamines. The combination of hard and soft segments allows polyurea to maintain its mechanical properties while enabling self-healing via chain diffusion. At temperatures above the glass transition temperature, the soft segment turns into the rubbery state. The chain mobility is only restricted by the hard segment. Due to the soft and hard segments being thermodynamically incompatible, there is microphase separation [65]. The hard and soft segments can be separated in two different ways: phase-mixed and phase-separated. In a phase-mixed morphology, the soft segment is directly joined to the hard segment. However, in a microphase-separated morphology, the soft segment is distinctively excluded from the hard segment [66].

The microphase-separated polyurea structure tends to have comparatively inferior self-healing because of the insufficient dynamic interaction present in the hard segment. Hence, a phase-mixed morphology is preferred for high self-healing efficiency. However, when the hard segment penetrates the soft segment, this will hinder the mobility of the soft segment [66], which results in slower chain mobility and lower self-healing efficiency. Hence, there is a need to find the optimum extent of microphase separation so that there is a balance between dynamic interactions and chain mobility for the most efficient self-healing to take place.

To obtain phase-mixed morphology, a chain extender with dynamic bonds can be included to equip the hard segment with dynamic interactions. Table 5 shows some of the common chain extenders that are included in the hard segment to enhance dynamic interactions for better self-healing efficiency. The degree of microphase separation can be adjusted by changing the type and ratio of hard segments. [67].

By synthesizing polyurea using different diisocyanates with different ratios, the number of respective types of hydrogen bonds (different strengths) in the synthesized polyurea will be different. The presence of different types of hydrogen bonds in the hard segment will affect how well the microphase in polyurea is separated, which in turn affects the self-healing efficiency.

### 3.3. Time before Surfaces Are in Contact

The way in which bridges are formed across the interface between two fractured surfaces of polyurea can be split into different regimes according to the waiting time [68]. The waiting time is defined as the time before the two fractured surfaces are brought into contact. The formation of bridges can lead to either self-adhesion or self-healing. Self-adhesion occurs when there is a low concentration of open stickers at unfractured surfaces, and the number of bridges grows slowly. Self-healing occurs when the system is far from equilibrium as a lot of stickers appear, so the number of bridges grows quickly.

**Figure 7:**
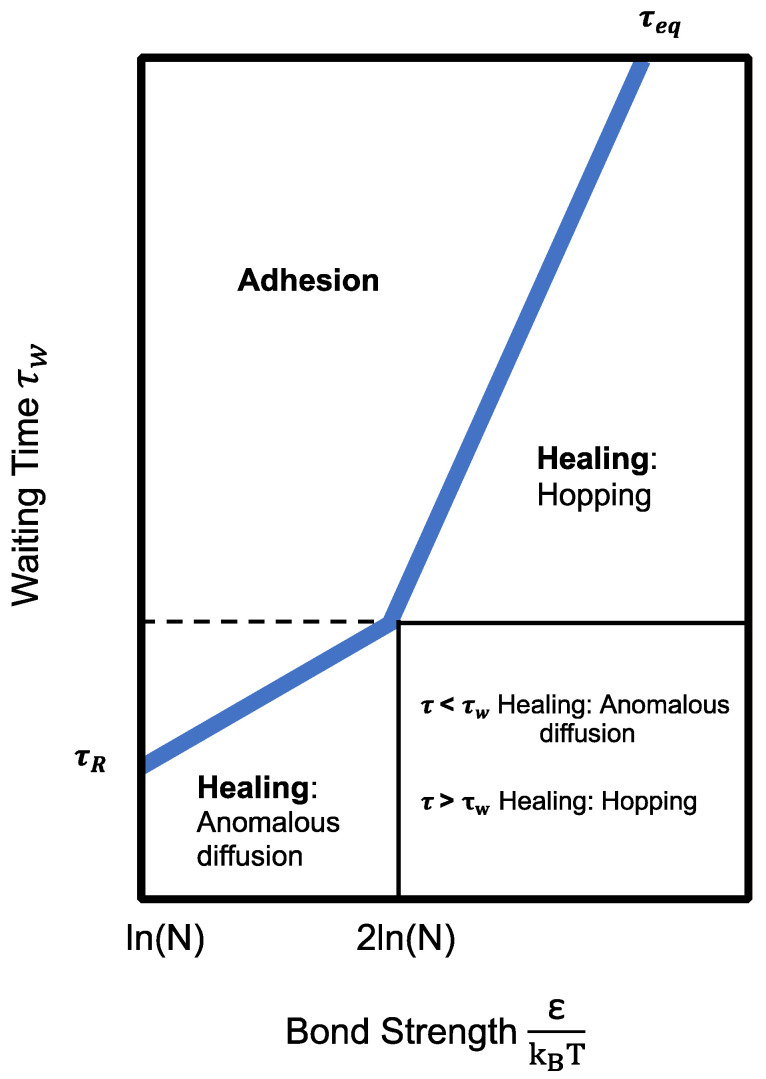
Graph showing regime of adhesion and healing ($\tau_{b}$ represents bond lifetime as a function of bond strength; $\tau_{eq}$ is the thick black line that represents bulk equilibrium time as a function of bond strength; $\tau_{R}$ represents rouse time).

According to Figure 7, self-healing occurs when the waiting time is shorter than the longest relaxation time of the suspension chain. With a short waiting time, there is a high concentration of open stickers at the fractured surfaces. To reduce the energy in the system, open stickers will recombine via anomalous diffusion and hopping to form bridges, which heal the fractured surfaces. As time increases, the open stickers may form loops with dangling chains, leading to a drop in the concentration of open stickers. The system will be close to equilibrium. Hence, self-adhesion occurs rather than self-healing. Therefore, the fractured surfaces have to be brought into contact within a short period of time to ensure that self-healing occurs. This is in line with Stage 2 (surface approach) of crack healing mechanisms.

### 3.4. Equilibrium Kinetics

Intrinsic mechanisms involve the use of dynamic bonds. Self-healing polyurea comprises dynamic bonds. Without them, polyurea will be a thermoset. Dynamic bonds allow forward reactions and backward reactions. However, this is different from reverse chemistry. Reverse chemistry does not always lead to dynamic properties [69]. For efficient self-healing, both the forward and backward reactions should be fast. This means that K and $K_{-1}$ should be large. However, $k_{1}$ should still be larger than $k_{-1}$. This is so that the formation of the polymer will be favored, and self-healing can occur to fill the crack. Hence, $K_{eq}$ ($K_{eq}=k_{1}$/$k_{-1}$) should also be large. Therefore, the reversibility of the reaction plays a role in enhancing the self-healing efficiency.

In order to obtain ideal equilibrium kinetics for high self-healing efficiency, the choice of chain extender for the synthesis of polyurea plays an important part. Different chain extenders have different equilibrium kinetics due to their structure and bulkiness. To obtain a high equilibrium constant, the amine used should not be bulky.

With reference to Table 5, the bulkiness of amine increases from diethylamine to tert-butyl-ethylamine to 2,2,6,6-Tertramethylpiperidine. Hence, polyurea made from 2,2,6,6-Tetramethylpiperidine has the lowest magnitude of K, which is too small to be applied for practical uses, as the polyurea will have a low degree of polymerization and very limited mechanical strength [70]. Comparing the values of $K_{eq}$ ($k_{1}$) and $K_{-1}$, TBEU has the highest magnitude for both and shows the best self-healing efficiency. Hence, the choice of chain extender will affect equilibrium kinetics.

## 4. Novel Strategy of Preparation of Self-Healing Polyurea

### 4.1. Choice of Isocyanate

Polyurea consists of a hard segment that includes isocyanate. Hence, the diisocyanate structure used to synthesize self-healing polyurea will have a significant impact on polyurea’s self-healing capability [66]. Table 6 shows the typical diisocyanate used in the synthesis of polyurea with high self-healing efficiency. IPDI has a bulky alicyclic structure, while HDI has a linear aliphatic structure. Comparing IPDI and HDI, IPDI has a bulkier structure than HDI. The bulky structure of IPDI prevents tight hard-segment packing. On the other hand, a hard segment with HDI will assemble into a tightly packed hard domain. As such, Yilgor et al. [71] reported that the tightly packed hard domain leads to the restriction of chain segmental motion. With a lower extent of chain mobility, the rate of Stages 1, 3, and 4 in crack healing mechanisms will be limited. This translates to lower self-healing efficiency. Similarly, HMDI also displays poor healing efficiency [72]. HMDI has strong hydrogen bonds that aggregate due to its rigid structure. The strong hydrogen bond is difficult to dissociate at room temperature. Therefore, chain mobility will also be restricted by HMDI and impede self-healing. Out of the three diisocyanates, IPDI shows the highest potential to form polyurea with the best self-healing efficiency.

### 4.2. Ratio of Different Isocyanates

As mentioned previously, the hard segment of polyurea consists of isocyanate. The structure of isocyanate used will affect segmental mobility, which in turn reduces the self-healing efficiency. The varying combinations of different isocyanates used in the hard segment will change the ratio between strong and weak hydrogen bonds [67], affecting the microphase separation and, eventually, the self-healing efficiency of polyurea. HMDI forms strong hydrogen bonds, while IPDI units tend to form weak hydrogen bonds. Hence, HMDI provides strong mechanical properties with weak self-healing capability, while IPDI is the opposite. However, Zhang [67] also reported that the presence of too few strong hydrogen bonds in IPDI could lead to instability of the complex structure and also decrease healing efficiency. Therefore, there is a need to find a balance for the ratio of hard segments for optimal self-healing polyurea equipped with acceptable mechanical properties for practical uses. Based on Zhang’s findings, an IPDI:HMDI ratio of 2:1 is the most suitable combination for self-healing polyurea.

### 4.3. Choice of Chain Extender

A chain extender (Table 7) can be added to the synthesis of polyurea to enhance self-healing efficiency. A comparison between polyurea containing chain extenders with and without disulfide was made. Polyurea consisting of a disulfide chain extender shows higher self-healing efficiency [65,73]. Although it is true that hydrogen bonds are effective in conferring self-healing capabilities, having both excessively large or small numbers of hydrogen bonds will have a negative impact on the effectiveness [60]. Having too many hydrogen bonds will lead to a rigid matrix with minimal chain mobility. Having too few hydrogen bonds may lead to a flowy matrix that has overly mobile chains, limiting the number of reactive groups available for the formation of bonds. There is an optimal number of hydrogen bonds required for optimal self-healing efficiency. Having a disulfide chain extender will substitute some of the hydrogen bonds in the hard segment with sulfide bonds instead. These sulfide bonds help to loosen the hard-segmental packing and promote higher self-healing efficiency.

On the other hand, bulky amines may result in faster dynamic covalent bonding [70]. This translates to the ability of bonds to undergo rapid repetitive breaking and formation, which enhances the self-healing of polyurea. However, amines have weak bond strength that may not be favorable for practical application. Hence, disulfide chain extenders such as AFD are the most suitable choice for efficient self-healing polyurea.

## 5. Conclusions and Position

Regarding the choice of diisocyanate, the papers available [66,67,73] were coherent in identifying IPDI as the diisocyanate, which is an important material for the synthesis of polyurea with high self-healing efficiency. Bulky alicyclic IPDI is able to prevent the hard segment from packing tightly, which could potentially hinder chain mobility. Since the important condition for improving the self-healing efficiency is the mobility of the chains, IPDI is a suitable candidate to be used to find the optimal formulation for the high self-healing efficiency of polyurea.

Regarding the ratio of diisocyanates, a ratio of IPDI to HMDI equal to 2:1 has been identified to be the best ratio for the synthesis of polyurea with high self-healing efficiency. Zhang used a bulky amine as a chain extender and experimented with different ratios of IPDI to HMDI. Eventually, he found the best ratio of the different diisocyanates used. This shows that the ratio of diisocyanates is a factor that can also affect the self-healing efficiency of polyurea.

Regarding the choice of chain extender, the papers [66,73] available were coherent in identifying that chain extenders with disulfides are the best option. Amine chain extenders without disulfide bonds with different structures and varying bulkiness were compared, but they were unable to provide self-healing capability with sufficient mechanical strength [69]. This shows that the presence of disulfide bonds in the chain extender plays an important role in achieving polyurea with high self-healing efficiency.

## Figures and Tables

**Table 1:** Explanation of the five stages of crack healing and their importance [48,49].

Stages	Definition
1. Surface rearrangement	The roughness or topography of the surface changes with external factors (pressure, time, and temperature).
2. Surface approach	Healing can only occur when surfaces are brought together, or a gap is filled with healing fluid.
3. Wetting	Surfaces have to wet each other and form an interface before healing can occur.
4. Diffusion	The most critical stage of strength development.
5. Randomization	Refers to the equilibration of the non-equilibrium conformations of chains near the surfaces.

**Table 2:** Summary and challenges of extrinsic and intrinsic mechanisms [14,30].

	Advantages	Disadvantages
Microcapsules (Extrinsic)	Capsules easily dispersed into matrix Simple healing concept	Limited to one healing cycle Solvents are toxic
Vascular network (Extrinsic)	Multiple healing cycles Healing agents evenly distributed	Blockage of core fiber during filling and releasing of healing fluid Matrix carrying healing fluid reduces composite strength
Dynamic bonds (Intrinsic)	Do not require any catalyst or healing agent Heal for infinite cycles	External stimuli to trigger healing

**Table 3:** Factors that affect the self-healing efficiency.

	Stages	Importance	Factors
Physical interaction (Intrinsic): 5-Stage crack healing	1. Surface rearrangement	Emphasizes the importance of T_g_ and relaxation time of surface molecules	Chain mobility
	2. Surface approach	Emphasizes the need for separated pieces to be in contact	Time before the surfaces are in contact
	3. Wetting	Rate determining with surface rearrangement	Chain mobility
	4. Diffusion	Affected by number of chains and number of monomers diffused	Chain mobility
	5. Randomization	Restoration of the molecular weight distribution and random orientation of chain segments	Equilibrium kinetics (chain extender used)
Chemical interaction (Intrinsic): Dynamic bonds		Bonds capable of undergoing repetitive breaking and reformation	Equilibrium kinetics (chain extender used)

**Table 4:** List of common diisocyanates.

Name	Abbreviation	Mw (g/mol)	Chemical Structure
4,4′-Diphenylmethanediisocyanate	MDI	250.25	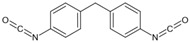
Toulene-2,4-diisocyanate	TDI	174.2	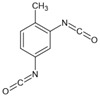
1,4-Butanediisocyanate	BDI	142.16	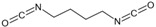

**Table 5:** List of chain extenders [70].

Chemical Name	Structure	Abbreviation of Polyurea Formed	$K_{eq}\left(M^{-1}\right)$	$K_{-1}\left(h^{-1}\right)$
2,2,6,6-Tetramethylpiperidine	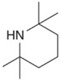	TMPCA	88	-
tert-Butyl-ethylamine	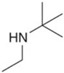	TBEU	$7.9\times 10^{5}$	0.21
Diethylamine	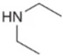	DEU	$>10^{7}$	0.0011

**Table 6:** List of diisocyanates.

Diisocyanate	Abbreviation	Chemical Structure
Isophorone diisocyanate	IPDI	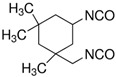
Hexamethylene diisocyanate	HDI72	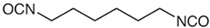
4,4-Dicyclohexylmethane diisocyanate	HMDI	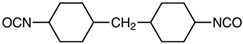

**Table 7:** List of chain extenders.

Chemical Name	Abbreviation	Chemical Structure
Bis(4-aminophenyl) disulfide	AFD	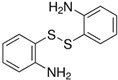
4,4′-Diaminodibenzyl	MDA	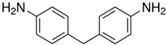

## Data Availability

All data related to this study are publicly available upon reasonable request to the first author.
